# Potential therapeutic target genes for systemic lupus erythematosus: a bioinformatics analysis

**DOI:** 10.1080/21655979.2021.1939637

**Published:** 2021-06-27

**Authors:** Yun Yu, Liang Liu, Long-Long Hu, Ling-Ling Yu, Jun-Pei Li, Jing-an Rao, Ling-Juan Zhu, Qian Liang, Rong-Wei Zhang, Hui-Hui Bao, Xiao-Shu Cheng

**Affiliations:** a Department of Cardiovascular Medicine, Second Affiliated Hospital of Nanchang University, Nanchang, Jiangxi, China; b Department of Rehabilitation, Second Affiliated Hospital of Nanchang University, Nanchang, Jiangxi, China; c Department of Rheumatology, Second Affiliated Hospital of Nanchang University, Nanchang, Jiangxi, China

**Keywords:** Systemic lupus erythematosus, bioinformatics, differentially expressed genes, tissue-specific gene expression, biomarkers

## Abstract

Systemic lupus erythematosus (SLE) is a chronic autoimmune disease involving multiple organs. However, the underlying etiology and mechanisms remain unclear. This study was performed to identify potential therapeutic targets for SLE using bioinformatics methods. First, 584 differentially expressed genes were identified based on the GSE61635 dataset. Tissue-specific analyses, enrichment analyses, and Protein–Protein interaction network were successively conducted. Furthermore, ELISA was performed to confirm the expression levels of key genes in the control and SLE blood samples. The findings revealed that tissue-specific expression of markers of the hematological system (25.5%, 28/110) varied significantly. *CCL2, MMP9*, and *RSAD2* expression was markedly increased in the SLE samples compared with controls. In conclusion, the identified key genes (*CCL2, MMP9*, and *RSAD2*) may act as possible therapeutic targets for the treatment of SLE.

## GRAPHICAL ABSTRACT

**Figure:**
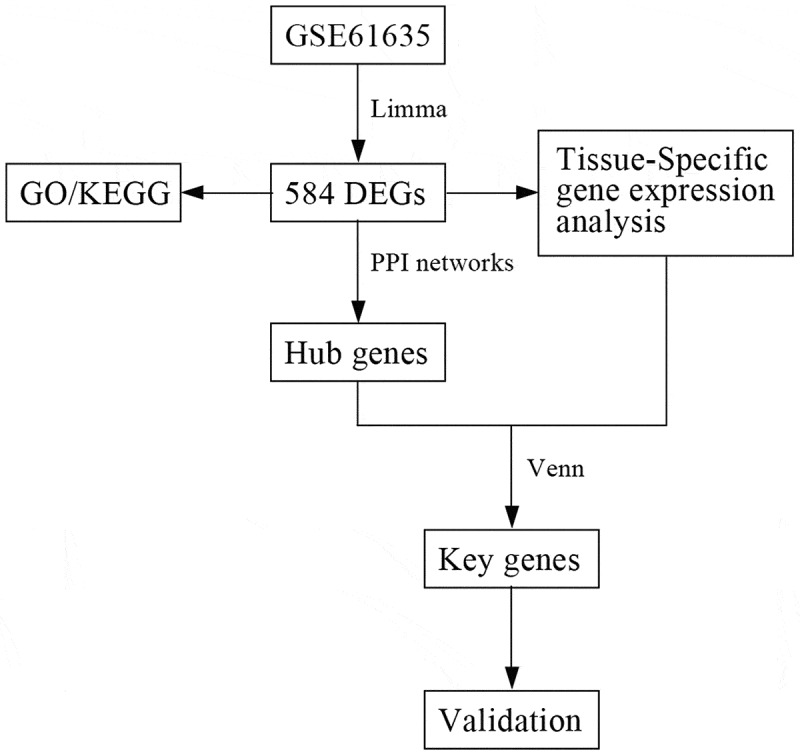

## Highlights

● Tissue-specific expression of haematological system markers varies significantly.

● CCL2, MMP9, and RSAD2 have potential therapeutic value in the treatment of SLE.

● Seven signalling pathways are positively related to SLE.

## Introduction

Systemic lupus erythematosus (SLE) is a polysystemic autoimmune disease involving multiple organs [1]. Epidemiological studies have suggested that the 10-year survival rate of patients with SLE is 90%, and 25% of deaths are caused by thrombotic events or concurrent infections [2–4]. Decades of research have revealed that genetic, immune, and environmental factors participate in the pathogenesis of SLE [5–8]. However, the precise pathogenic mechanisms underlying SLE remain to be fully elucidated. Currently, there is no cure for SLE, and the treatment mostly relies on nonsteroidal anti-inflammatory drugs (NSAIDs) and immunosuppressants to relieve symptoms.

The Gene Expression Omnibus (GEO) database contains gene profiles generated predominantly using DNA microarray technology [9,10]. This study aimed to explore the potential hub genes and underlying mechanisms in SLE using bioinformatics methods. Raw data from microarray analyses conducted on SLE samples and healthy controls were downloaded from the GEO database. According to the enrichment analysis, BioGPS, String database, and protein–protein interaction (PPI) network analysis were utilized to identify key genes. By verifying the selected key genes, the validation results provide a basis upon which novel insights regarding mechanisms underlying SLE and new approaches for SLE therapeutic intervention can be developed.

## Materials & methods

### Data source

Microarray dataset GSE61635 was available at the Gene Expression Omnibus (GEO) database (www.ncbi.nlm.nih.gov/geo/). It was based on the GPL570 platform (HG-U133_Plus_2), comprising 99 SLE blood samples and 30 healthy control samples.

### Data processing

Raw data were processed and analyzed using R (version 4.0.2). The median value of each sample was normalized using the limma package between arrays for background correction. A robust multichip average (RMA) was then created, and perfect matches from the raw data were log-transformed. FDR <0.05 and |log2 fold change (FC)| >1 were considered for the differentially expressed genes (DEGs) [11]. DEGs were processed and plotted as volcano plots and a heatmap using ggplot2 and pheatmap R packages, respectively.

### Tissue-specific gene expression analysis

Information regarding the function of a gene can be obtained from the relative tissue-specific genes. To screen out tissue-specific DEGs, the BioGPS database (http://biogps.org/#goto=welcome) was used [12]. Highly tissue-specific transcripts mapped to a single tissue were included if all of the following criteria were met: (a) median expression > 30 times the median expression of all other tissues; (b) the highest expression level was at least threefold higher than the second-highest expression.

### Functional annotation and KEGG pathway analysis

GO [13] and KEGG [14] pathway analysis of DEGs were screened out by using DAVID 6.8 (http://david.abcc.ncifcrf.gov/) online database [15]. Significant difference was set at *P*< 0.05.

### Identification of key genes

STRING (https://string-db.org/) was used to construct the PPI network [16]. The confidence score was set at ≥0.4. Cytoscape v3.7.2 and the CytoHubba plugin (version 0.1) were used to visualize and identify the PPI network. The top 20 hub genes were obtained based on the filtering algorithm (closeness). A Venn diagram was then delineated to confirm the key genes between hub genes and tissue-specific genes.

### ELISA

The experimental protocol was approved by the Ethics Committee of the Second Affiliated Hospital of Nanchang University in compliance with the Declaration of Helsinki. SLE and normal subjects were informed of the study content in oral form. Two milliliters of blood was collected and anticoagulated with EDTA. Serum samples were collected by centrifuging the blood samples at 2000 rpm for 10 min at 4°C. All ELISA kits (CCL2, MMP9, GATA1, and RSAD2) were used according to the manufacturer’s instructions (MEIMIAN, Jiangsu Biological Industrial Co., Ltd., China).

### Statistical analysis

A minimum of three replicates were performed for each experiment, and data are presented as the mean ± SD. Statistical analyses were performed using GraphPad Prism 8 (GraphPad Software, San Diego, USA). Comparisons between groups were performed using an unpaired *t*-test. Statistical significance was set at *p*< 0.05.

## Results

In order to explore potential therapeutic targets of SLE, bioinformatics methods were used to identify DEGs. We next performed tissue-specific gene expression analysis and enrichment analysis and constructed a PPI network. Finally, the selected hub genes were verified using ELISA. Therefore, this study may significantly improve the targeted therapy of SLE and enrich our understanding of its pathogenesis.

### Differential expression analysis

**Figure 1.**
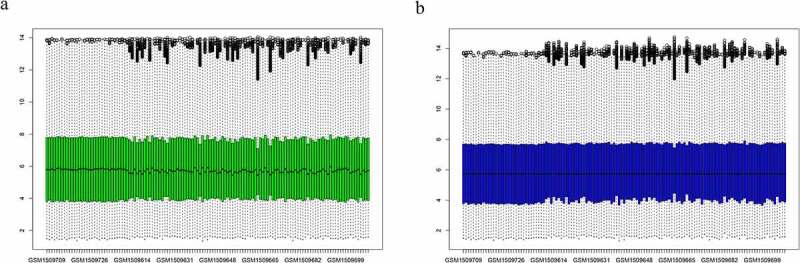
Normalization of microarray dataset. (a) Before normalization of the GSE61635 dataset. (b) After normalization of the GSE61635 dataset

**Figure 2.**
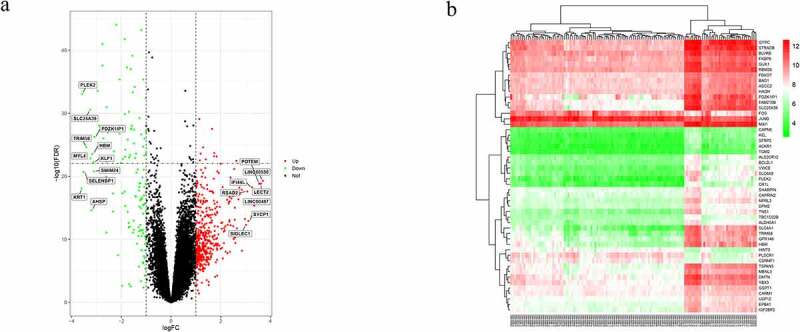
Differentially expressed genes (DEGs) between systemic lupus erythematosus (SLE) and control groups. (a) Volcano plot of GSE61635; 19 significantly expressed genes were identified. Red, green, and black dots represent upregulated, downregulated, and unchanged genes, respectively. (b) Heatmap of the top 50 DEGs from GSE61635

In total, 99 patients with SLE and 30 normal subjects were enrolled in this study. Microarray data of the GSE61635 dataset were standardized (Figure 1). After setting the cutoff at FDR <0.05, and |log2 (FC)| >1, 584 DEGs were identified (Figure 2). The 19 significantly expressed genes between the two groups were extracted using cutoffs at |log2 (FC)| >3 and FDR <0.005 (Figure 2(a)).

### Specificity of DEGs in tissue expression

Using BioGPS, we screened 110 DEGs that were preferentially expressed in specific tissues. Tissue-specific expression of the markers of the hematological system (25.5%, 28/110) varied significantly, followed by the urinary/genital system (22.7%, 25/110), neurologic and digestive system (18.2%, 20/110), respiratory and skin/skeletal muscle system (10.9%, 12/110), immune system (9.1%, 10/110), endocrine system (7.3%, 8/110), and circulatory system (4.5%, 5/110) (Table 1).

**Table 1.** Tissue-specific genes identified by BioGPS

System	Genes
Hematological	PARP9, KLF2, C15orf48, RSAD2, CYSLTR1, INHBA, AHSP, GYPB, CXCR4, LILRA5, NRBF2, GYPE, GAGE1, SPTA1, CEBPD, HSPA6, BACH2, UCHL1, TMEM140, ANXA3, CLIC2, FPR2, FFAR2, BPGM, PLOD2, HCAR3, FCRLA, EIF1AY
Immune	FBXO7, EPSTI1, SLC14A1, LILRA4, CXCR4, PALM2AKAP2, CPA3, FCRLA, CD160, KDM5D
Neurologic	ALDH5A1, RUNDC3A, SLC17A6, ERBB4, TSPAN7, PMP2, UCHL1, SOX11, NAP1L3, PEG3, TAC1, RPH3A, TSHB, GRM3, IFI27, OPCML, LIMCH1, TUBB2A, ECRG4, NRN1
Skin/Skeletal muscle	TNFAIP6, PRTG, G0S2, GATA1, EPB42, ANK1, HMBS, AHSP, GYPB, CA1, MMP8, MMP9
Respiratory	FLACC1, ADM, FAT1, P4HA2, F3, ODAM, ANXA3, JUP, IFI27, TCN1, SLPI, CAV1
Digestive	CTSE, GYPB, G0S2, SPTA1, GIPC2, GIPC2, PEG3, ANXA3, UGT2B28, DSP, CLCA1, MBL2, TSPAN8, TDO2, CA1, CLCA4, CAV1, HP, HPR, OLFM4, APOBEC3B
Circulatory	G0S2, UCHL1, CCL2, PLOD2, CAV1
Urinary/Genital	PDZK1IP1, SMIM24, TRIM6, KRT1, CXCR4, GAGE1, KCNJ16, RNASEH2A, TGM2, POTEM, DHX58, AHSP, GYPB, GMPR, FAP, ADM, TPTE, MEIS2, DSP, CCNA1, SLC26A8, TSPAN8, PAEP, CABS1, CD177
Endocrine	KCNJ16, OPRPN, ECRG4, PLOD2, TCN1, SLPI, SMR3A, CRISP3

### Enrichment analysis of DEGs

**Figure 3.**
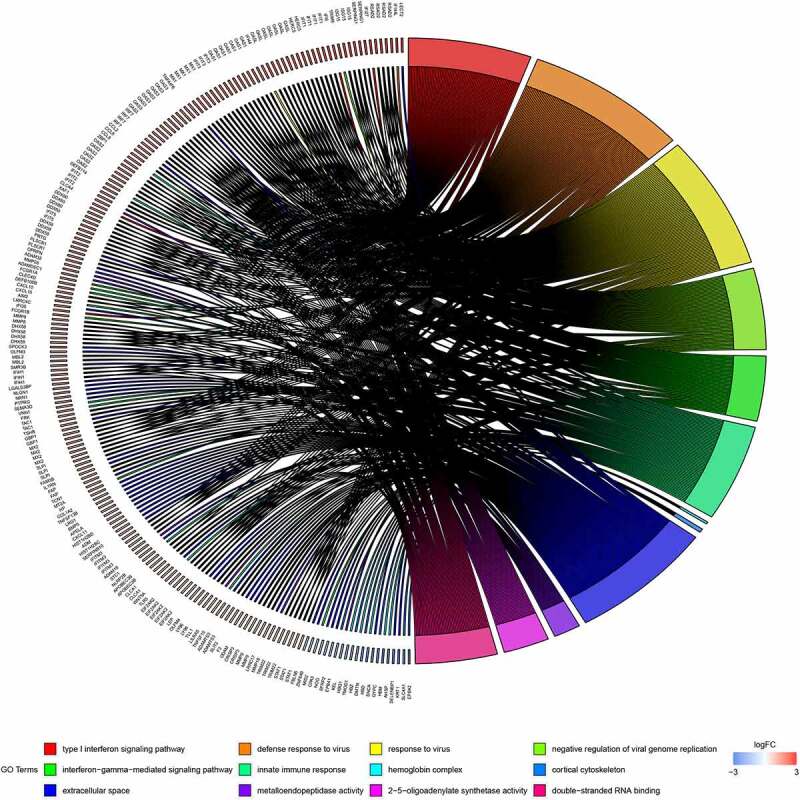
Distribution of differentially expressed genes (DEGs) in systemic lupus erythematosus (SLE) for GO enrichment

**Figure 4.**
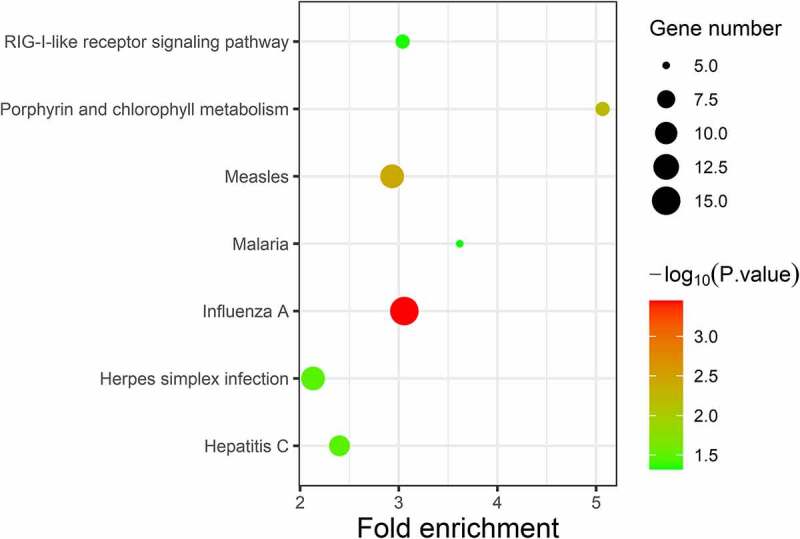
KEGG enrichment analysis of differentially expressed genes (DEGs). Strength of the color represents the *p*-value (from the lowest in green to the highest in red), and the bubble size represents the number of DEGs

GO analysis was conducted using the DAVID software. Enriched GO terms were divided into three categories: BP, CC, and MF. As shown in Table 2 and Figure 3, the DEGs were mainly enriched in the ‘Type I interferon signalling pathway’ and ‘Defence response to virus’ in the BP group. CC analysis indicated that the DEGs were mainly enriched in ‘hemoglobin complex’ and ‘cortical cytoskeleton’. In terms of MF, DEGs were most enriched in ‘metalloendopeptidase activity’ and ‘2ʹ-5ʹ-oligoadenylate synthetase activity’. Pathway enrichment analysis of DEGs using the KEGG. KEGG analysis of DEGs revealed that they were mainly enriched in ‘influenza A’, ‘measles’, and ‘porphyrin and chlorophyll metabolism’ (Table 3 and Figure 4).

**Table 2.** GO analysis of significant DEGs in SLE

Category	Term	Genes	FDR
**BP**	Type I interferon signaling pathway	IFITM3, RSAD2, STAT1, MX2, MX1, IFI6, ISG15, IFI35, IFIT1, IFIT3, IFIT2, OASL, IFI27, OAS1, OAS2, OAS3, IRF7, XAF1	1.20E-09
	Defense response to virus	IFITM3, RSAD2, STAT1, MX2, MX1, IFIT5, EIF2AK2, ISG15, IFIT1, DDX60, IFIT3, IFI44L, IFIT2, OASL, HERC5, CXCL10, PLSCR1, OAS1, OAS2, OAS3, DHX58, GBP1, TRIM22, APOBEC3B	6.20E-08
	Response to virus	IFITM3, RSAD2, DDX58, MX2, MX1, IFI44, EIF2AK2, IFIT1, DDX60, IFIT3, IFIT2, OASL, IFIH1, CCL8, OAS1, OAS2, OAS3, DHX58, IRF7, TRIM22	6.20E-08
	Negative regulation of viral genome replication	IFITM3, TRIM6, PLSCR1, RSAD2, OAS1, SLPI, OAS3, MX1, EIF2AK2, ISG15, IFIT1, OASL	2.69E-06
	Interferon-gamma-mediated signaling pathway	MT2A, OAS1, STAT1, OAS2, OAS3, IRF7, FCGR1A, FCGR1B, GBP1, TRIM22, OASL	0.007775401
	Innate immune response	CRISP3, IFIT5, LY96, DDX60, LILRA5, IFIH1, HERC5, DHX58, TAC1, MBL2, ZBP1, NLRP2B, DDX58, MX2, MX1, DEFB114, EIF2AK2, DEFB108B, MID2, CLEC4D, AIM2, VNN1, SLPI, IRF7, SERPING1, TLR5, FRK, APOBEC3B	0.013089362
**CC**	Hemoglobin complex	HBZ, HBM, AHSP, HBD, HBQ1	0.023035163
	Cortical cytoskeleton	TMOD1, DMTN, GYPC, EPB42, EPB41, SLC4A1	0.023035163
	Extracellular space	LGALS3BP, IL1RN, C2ORF40, TNFAIP6, CRISP3, LECT2, HP, ADM, PTPRG, TNFSF13B, SMR3B, ADAMTS3, ZNF649, TNFSF10, FAM3B, TSHB, MBL2, CPA3, KRT1, NOG, WNT5A, OLFM4, MMP8, MMP9, F3, OPRPN, SFRP2, OLFM3, SLPI, OAS3, SERPING1, HIST1H2BD, HIST1H2BC, SERPINB10, NLGN1, NRN1, SEMA3D, LRRC17, LY96, STC1, C9ORF72, FBLN5, PRTG, SELENBP1, APELA, CCL8, SPOCK3, CLCA1, CCL2, SLIT2, TAC1, LRRC4C, SNCA, ODAM, BMP5, CXCL10, CXCL11, LRG1, COL1A2, FAP, TCN1, LEP	0.004656585
**MF**	Metalloendopeptidase activity	ADAMDEC1, ADAM32, MMP8, MMP9, ADAM18, ADAMTS3, KEL, MMP16, FAP, MMP26, CLCA1, TLL1, CLCA4	0.021510128
	2ʹ-5ʹ-oligoadenylate synthetase activity	OAS1, OAS2, OAS3, OASL	0.021510128
	Double-stranded RNA binding	IFIH1, OAS1, DDX58, OAS2, OAS3, DHX58, EIF2AK2, DDX60, OASL	0.037427071

**Table 3.** KEGG pathway analysis of significant DEGs in SLE

Pathway ID	Name	Genes	P-Value
hsa05164	Influenza A	RSAD2, DDX58, STAT1, HSPA6, MX1, EIF2AK2, NXT2, IFIH1, CXCL10, OAS1, OAS2, OAS3, TNFSF10, IRF7, CCL2	3.55E-04
hsa05162	Measles	IFIH1, OAS1, DDX58, STAT1, OAS2, OAS3, MX1, HSPA6, IRF7, TNFSF10, EIF2AK2	0.004053153
hsa00860	Porphyrin and chlorophyll metabolism	ALAS2, HEPH, FECH, HMBS, BLVRB, UGT2B28	0.006044328
hsa05168	Herpes simplex infection	IFIH1, OAS1, DDX58, STAT1, OAS2, OAS3, IRF7, EIF2AK2, CCL2, FOS, IFIT1	0.033041831
hsa05160	Hepatitis C	OAS1, DDX58, STAT1, OAS2, CLDN8, OAS3, IRF7, EIF2AK2, IFIT1	0.033050818
hsa04622	RIG-I-like receptor signaling pathway	IFIH1, CXCL10, DDX58, DHX58, IRF7, ISG15	0.046053009
hsa05144	Malaria	GYPA, GYPC, GYPB, CCL2, ACKR1	0.047754191

### PPI network construction and key genes identification

**Figure 5.**
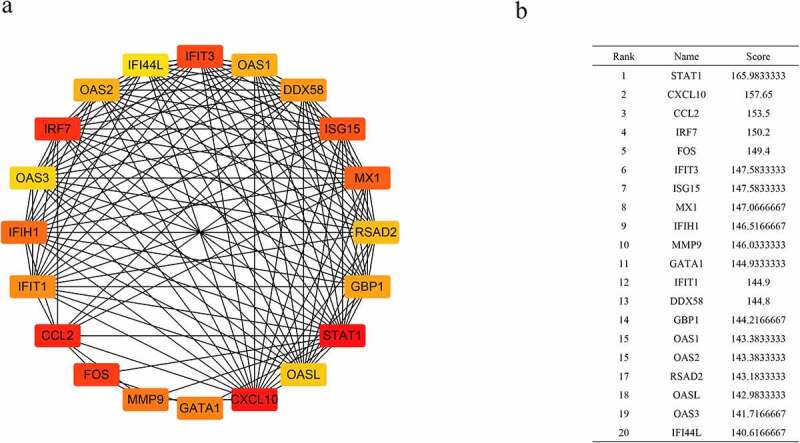
The PPI network of differentially expressed genes (DEGs). (a) Top 20 DEGs visualized based on the Closeness algorithm analysis in Cytoscape. (b) Top 20 DEGs based on Closeness score ranking

**Figure 6.**
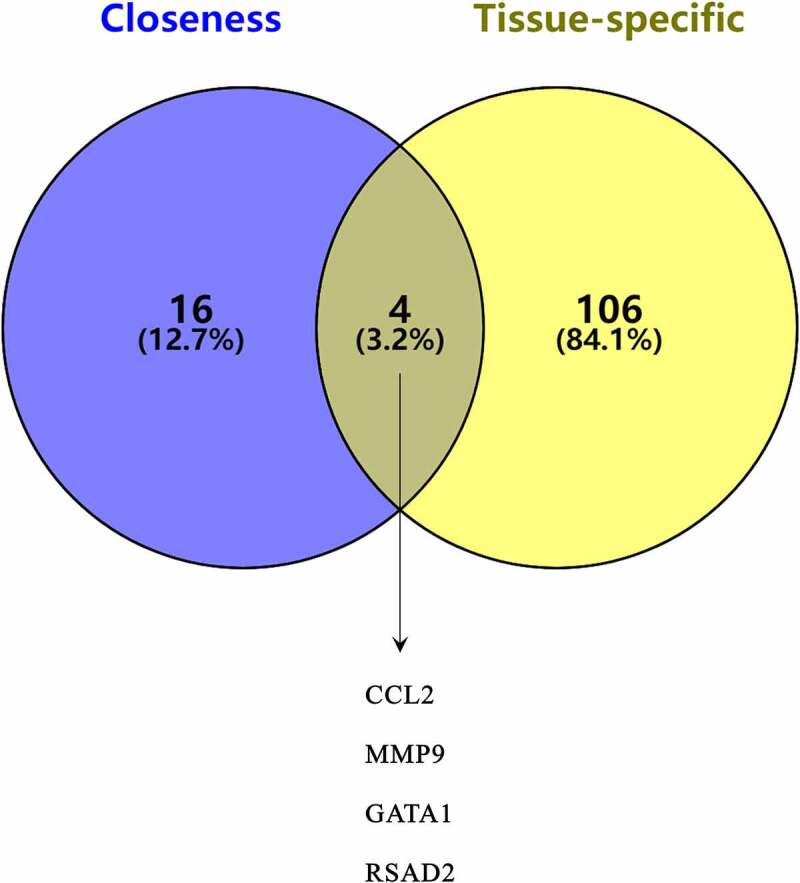
Key genes between hub genes and tissue-specific genes

Using the STRING database and Cytoscape v3.7.2, a PPI network was constructed. Based on the closeness algorithm, the top 20 hub genes were identified using the Cytohubba plugin. Hub genes were extracted, and the top 20 connected proteins were shown together with the rank of each hub gene, including *STAT1, CXCL10, CCL2, IRF7, FOS, IFIT3, ISG15, MX1, IFIH1, MMP9, GATA1, IFIT1, DDX58, GBP1, OAS1, OAS2, RSAD2, OASL, OAS3*, and *IFI44L* (Figure 5). Next, a Venn diagram was constructed to confirm the key genes between hub genes and tissue-specific genes (Figure 6).

### Validation of gene expression

**Figure 7.**
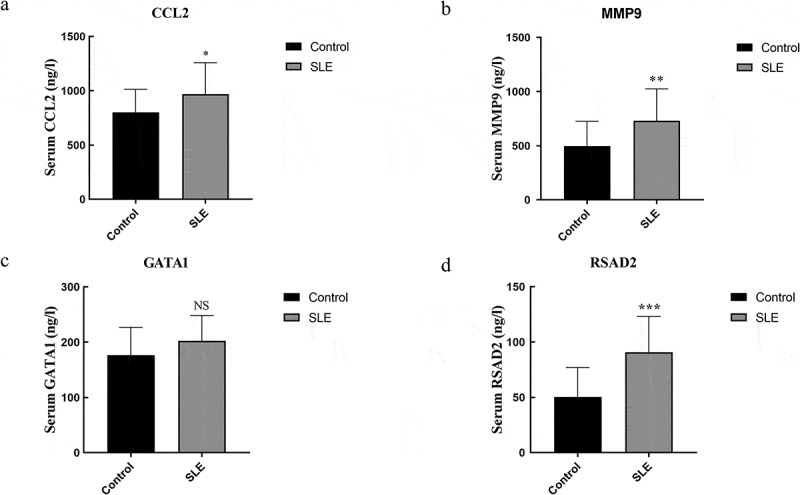
Concentration of CCL2, MMP9, GATA1, and RSAD2 in the serum. (a–d) Concentration of CCL2, MMP9, GATA1, and RSAD2 in the serum. Data are presented as mean ± SD (n ≥ 15). * *p* < 0.05, **p < 0.01, *** *p*< 0.005 vs Control; NS, no statistical significance vs Control

Expression of four key genes (*CCL2, MMP9, GATA1*, and *RSAD2*) was verified using ELISA in control and SLE subjects. The ELISA results showed that the levels of *CCL2, MMP9*, and *RSAD2* in the SLE group were significantly increased (Figure 7).

## Discussion

Previously, a significant number of genes have been shown to correlate with SLE [17,18]. However, current therapies for SLE have limited efficacy and increased susceptibility to secondary outcomes [19]. In this study, 584 DEGs were obtained from the selected dataset GSE61635. Enrichment analysis of DEGs showed that they were primarily involved in the hemoglobin complex, immune response, and metalloendopeptidase activity. Compared with previous researches, we conducted a tissue-specific analysis of differential gene expression, which could potentially allow for the development of more effective and targeted therapeutics [20,21]. The results suggested that 110 DEGs were involved in the hematological system, urinary/genital system, neurologic and digestive system, respiratory and skin/skeletal muscle system, immune system, endocrine system, and circulatory system. Furthermore, four key genes were revealed between hub genes and tissue-specific genes, including *CCL2, MMP9, GATA1*, and *RSAD2*. The statistical results validated by ELISA showed that the levels of CCL2, MMP9, and RSAD2 were significantly increased in the SLE group.

Chemokines are a family of small peptides that are involved in cell trafficking and inflammatory responses [22–24]. Currently, approximately 50 different chemokines have been identified, most of which belong to the CC and CXC families [25]. Monocyte chemoattractant protein-1 (MCP-1 or CCL2), a prototype of the CC subfamily, plays a crucial role in inflammatory processes [26,27]. CCL2/MCP-1 is significantly correlated with SLE, and CCL2 levels are significantly reduced after treatment [28,29]. Moreover, it has been demonstrated that CCL2/MCP-1 is strongly associated with atherosclerosis and cardiovascular diseases (CVD) in patients with SLE [30,31].

Matrix metalloproteinases (MMPs), also known as matrixins, are extracellular matrix (ECM)-degrading enzymes [32]. MMP-9, an extracellular proteinase, is involved in various pathophysiological processes, such as ECM remodeling, inflammatory response, and immune response [33]. Multiple cytokines play crucial roles in upregulating the expression of MMP-9 in response to inflammation [34]. However, MMP-9 appears passively as a downstream product of the inflammatory response. Additionally, it plays a positive feedback role on many pro-inflammatory factors (IL-1β and IL-8), which are important ‘regulators’ of the inflammatory response [35]. Prior studies have shown that MMP-9 plays a significant role in chronic autoimmune diseases, such as SLE, by activating the inflammatory response [36,37]. MMP-9 degrades components of the vascular basement membrane that help inflammatory cells invade the vascular wall and induce inflammation associated with the pathogenesis of SLE, thus increasing endothelial cell permeability [38,39].

*RSAD2*, an interferon-inducible gene, is involved in the innate immune response against viruses [40,41]. RSAD2 activates the immune response and has been associated with multiple autoimmune diseases, such as RA, SLE, and AS [42,43]. Doedens et al. [44] found that patients with SLE have an important link with IFN dysregulation. A study performed by Sezin et al. [42] showed that RSAD2 is the hub gene in the pathogenesis of SLE.

There are several limitations to this study. First, it was performed at a single center in China; therefore, the results warrant further validation in other populations. Second, only one dataset was utilized in this study, and future studies will be required to validate these findings in other datasets. Further large-scale validation studies and molecular mechanisms of SLE should be performed to explore the roles of these genes.

## Conclusion

In conclusion, the present investigation demonstrates that *CCL2, MMP9*, and *RSAD2* are linked to the initiation and development of SLE. These genes and the related pathways may serve as novel therapeutic targets for SLE. Large-scale, multi-center research is needed to further validate these findings.

## Data Availability

The GSE61635 dataset analysed in this study was downloaded from the NCBI GEO database.

## Author statement

Yun Yu: Statistical Analysis, Manuscript Preparation. Liang Liu: Data Collection, Statistical Analysis. Long-long Hu, Jun-pei Li, Ling-ling Yu, Ling-juan Zhu and Qian Liang: Data Interpretation. Jing-an Rao and Rong-wei Zhang: Data Collection. Hui-hui Bao and Xiao-shu Cheng: Study Design and Manuscript revision.
